# Recent Advancements in the Management of Anti-neutrophil Cytoplasmic Antibody-Associated Vasculitis: A Systematic Review

**DOI:** 10.7759/cureus.21814

**Published:** 2022-02-01

**Authors:** Hadia Arzoun, Mirra Srinivasan, Santhosh Raja Thangaraj, Siji S Thomas, Alena Yarema, Bridget Lee, Lubna Mohammed

**Affiliations:** 1 Internal Medicine, California Institute of Behavioral Neurosciences & Psychology, Fairfield, USA; 2 Internal Medicine, St. Bernards Medical Center, Jonesboro, USA

**Keywords:** rituximab, complement, plasma exchange, monoclonal antibodies, glucocorticoids, treatment modalities, recent advancements, anca, anti-neutrophil cytoplasmic antibody-associated vasculitis

## Abstract

Anti-neutrophil cytoplasmic antibody (ANCA)-associated vasculitis is a rare multisystem autoimmune condition that causes inflammation of small and medium-sized blood vessels and is more commonly seen in the geriatric population. ANCA-associated vasculitis (AAV) is typically characterized as necrotizing vasculitis and includes granulomatosis with polyangiitis (GPA), microscopic polyangiitis (MPA), and eosinophilic granulomatosis with polyangiitis (EGPA). The mortality rate remains high, with especially cardiovascular disease, infections, and malignancies being the leading causes of death. Existing treatment options depend heavily on the use of glucocorticoids (GCs), often in combination with cyclophosphamide (CYC); however, as the multitude of adverse effects associated with these agents has increased, numerous studies are being conducted to reduce not only these harmful effects but also improve remission rates. Rituximab, avacopan, corticosteroids, including intravenous pulse methylprednisolone, plasma exchange, and immunological targeting are among the emerging treatments. The purpose of this review is to emphasize the pathogenesis and traditional treatment modalities and give insights into the recent advances in managing this disorder in an attempt to spare the adverse effects of conventional therapies while achieving better remission rates with combination therapies as well. The authors explored multiple databases, employing appropriate keywords, satisfying the quality appraisal, after which a total of 14 reports were included in this review. Upon overall analysis, it can be concluded that rituximab and CYC, when used in combination, provided a safer alternative to GCs while exhibiting equal, if not superior, effectiveness and results, thus, paving the way for additional in-depth research in a larger population of interest.

## Introduction and background

Vasculitis is a disorder in which the blood vessels become inflamed [1], resulting in vessel wall thickening and a reduction in the amount of blood that can flow through them [2]. Anti-neutrophil cytoplasmic antibody (ANCA)-associated vasculitis is a severe and chronic condition that affects the small blood vessels in the body and is characterized by autoantibodies that target neutrophils. Leukocyte proteinase 3 (PR3) and myeloperoxidase (MPO) are the two primary antigens targeted by ANCAs [3], which are found on the membranes of activated neutrophils and monocytes [4]. The etiology of ANCA-associated vasculitis (AAV) has remained multifactorial and is thought to be affected by factors such as genetics, environmental conditions [1], infections [4], and innate/acquired immunity [1]. Though relatively rare in its occurrence, the condition traditionally faced poor prognosis [3] as the immune cells infiltrate and subsequently damage small and medium-sized blood arteries [4]. Some of the clinical phenotypes of AAV include granulomatosis with polyangiitis, microscopic polyangiitis, eosinophilic granulomatosis with polyangiitis, and renal-limited vasculitis [1]. Long-established treatment options relied on glucocorticoids (GCs), often in combination with cyclophosphamide (CYC) [4]. However, recent advancements in the treatment and management of AAV have dramatically increased patient prognosis in recent years [3], including the use of rituximab, avacopan, plasma exchange, immunological targeting, and intravenous pulse methylprednisolone, with all demonstrating success in achieving remission [2,4-9]. Additionally, effective contemporary treatment involves combining plasma exchange with GCs, rituximab with CYC, and rituximab with GCs [9-16]. The purpose of this review is to provide relevant background data regarding the prevalence, significance, pathophysiology of AAV, and traditional treatment modalities while also describing the contemporary advancements in this disorder's treatment and management capabilities.

Methodology

A literature search was done on the PubMed, Google Scholar, Science Direct, and Cochrane Library databases using regular and medical subject heading (MeSH) keywords through the Boolean scheme, as listed below. The inclusion criteria are set as full-text reports from the last five years, in the English language, across the globe, with study designs of observational studies and review articles. Reports older than 2016, non-full-text and non-English reports, and other study designs, such as randomized controlled trials, were excluded. All retrieved reports underwent a quality screening using the appropriate quality assessment tools. The authors of this systematic review followed the preferred reporting items for systematic reviews and meta-analysis (PRISMA) 2020 guidelines and principles [17].

Keywords

MeSH Keywords

ANCA Vasculitis OR (((( "Anti-Neutrophil Cytoplasmic Antibody-Associated Vasculitis/complications"[Mesh] OR "Anti-Neutrophil Cytoplasmic Antibody-Associated Vasculitis/drug therapy"[Mesh] OR "Anti-Neutrophil Cytoplasmic Antibody-Associated Vasculitis/epidemiology"[Mesh] OR "Anti-Neutrophil Cytoplasmic Antibody-Associated Vasculitis/etiology"[Mesh] OR "Anti-Neutrophil Cytoplasmic Antibody-Associated Vasculitis/pathology"[Mesh] OR "Anti-Neutrophil Cytoplasmic Antibody-Associated Vasculitis/prevention and control"[Mesh] )) AND treatment option OR steroids OR rituximab OR recent advancements OR "Glucocorticoids/therapeutic use"[Mesh]) OR "Plasma Exchange/therapy"[Mesh]) OR "Antibodies, Monoclonal/therapeutic use"[Mesh]

Keywords on Other Databases

Anti-Neutrophil Cytoplasmic Antibody-Associated Vasculitis; ANCA; recent advancements; treatment modalities; glucocorticoids; monoclonal antibodies; plasma exchange; complement; rituximab

Quality assessment

Table 1 depicts the type of study reviewed and corresponding scores awarded to each study using the appropriate quality appraisal tools [5-14,18-22].

**Table 1:** Summary of Study Designs and Corresponding Scores Awarded to Each Study SANRA: Scale for the assessment of narrative review articles

Author	Year	Type of Study	Level of Evidence	Quality Appraisal Tool	Scores
Yates et al. [18]	2017	Review Article	V	SANRA Check-list	>9; Include
Cortazar et al. [10]	2017	Retrospective Cohort	IV	Newcastle-Ottawa	>10; Include
McAdoo et al. [13]	2018	Retrospective Cohort	IV	Newcastle-Ottawa	>10; Include
Arman et al. [19]	2018	Review Article	V	SANRA Check-list	>9; Include
Pepper et al. [14]	2018	Prospective Cohort	IV	Newcastle-Ottawa	>10; Include
Ennis et al. [20]	2019	Review Article	V	SANRA Check-list	>9; Include
Chanouzas et al. [5]	2019	Retrospective Cohort	IV	Newcastle-Ottawa	>10; Include
Fenoglio et al. [11]	2020	Review Article	V	SANRA Check-list	>9; Include
Neumann et al. [7]	2020	Review Article	V	SANRA Check-list	>9; Include
Floyd et al. [21]	2021	Retrospective Cohort	IV	Newcastle-Ottawa	>10; Include
Monti et al. [12]	2021	Review Article	V	SANRA Check-list	>9; Include
Samman et al. [9]	2021	Review Article	V	SANRA Check-list	>9; Include
Onuora et al. [8]	2021	Review Article	V	SANRA Check-list	>9; Include
Jain et al. [6]	2021	Review Article	V	SANRA Check-list	>9; Include
Anders et al. [22]	2021	Review Article	V	SANRA Check-list	>9; Include

Results

**Figure 1:**
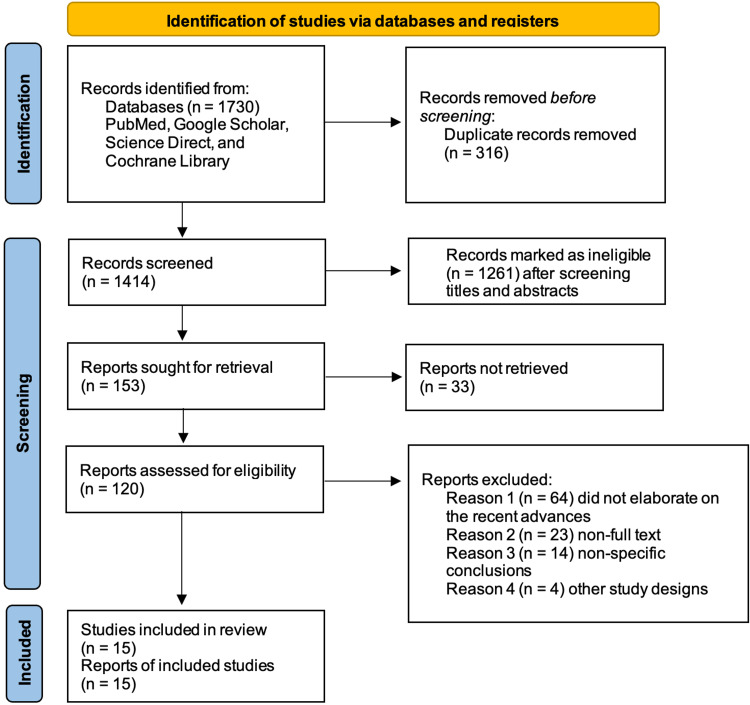
PRISMA Flow Chart 2020

A total of 1730 articles were found upon employing the appropriate keywords. After removing 316 duplicates before the screening, 1414 articles underwent the screening process where 1261 articles were removed based on their titles and abstracts. The authors retrieved 153 articles, where 33 were not retrievable and 120 reports were screened for eligibility. A final of 15 reports were included in the review upon an in-depth analysis of quality, inclusion/exclusion criteria, and study designs. The first and second authors conducted the data extraction and appraised the studies independent of each other; when a difference of opinion was raised, a third author was approached to meet common ground. The search strategy and the process of selecting the final studies included in this review are depicted in Figure 1 below in the form of a PRISMA flow diagram [17].

Table 2 below summarizes the results of the final included studies [5-14,18-22].

**Table 2:** Summary of the Conclusions for the Included Studies AAV: Anti-neutrophil cytoplasmic antibody-associated vasculitis; GCs: Glucocorticoids; CYC: Cyclophosphamide; MP: Methylprednisolone; ANCA: Anti-neutrophil cytoplasmic antibody; PR3-ANCA: Proteinase 3– ANCA; GPA: granulomatosis with polyangiitis; MPA: microscopic polyangiitis; PLEX: Plasma exchange; ESRD: End-stage renal disease; ADVOCATE: A Phase 3 Clinical Trial of CCX168 (Avacopan) in Patients With ANCA-Associated Vasculitis

Author	Conclusion
Yates et al. [18]	Most patients with AAV should get CYC or rituximab, in addition to GCs, as induction therapy. Rituximab should be investigated as an alternate induction drug for people at high risk of infertility and infection.
Cortazar et al. [10]	Combination therapy (rituximab plus CYC and an accelerated prednisone taper) was effective and well-tolerated, resulting in an early tapering of high-dose GCs.
McAdoo et al. [13]	Combination therapy (rituximab and CYC) was superior to the current standard of care.
Arman et al. [19]	Early detection and treatment can result in far better outcomes, particularly in retaining kidney function and preventing the need for renal replacement therapy. Despite the significant side effects, immunosuppressive (calcineurin inhibitors and CYC) medications remain the best option for reducing complications and improving results.
Pepper et al. [14]	Early GCs discontinuation in severe AAV is as effective as conventional therapy for remission induction and is associated with fewer GCs-related adverse effects.
Ennis et al. [20]	The existing evidence supports using mepolizumab for the induction and maintenance of remission in refractory, relapsing, or glucocorticoid-dependent EGPA, with ANCA-positive patients or those with increased eosinophilia possibly benefiting more.
Chanouzas et al. [5]	Incorporating intravenous pulse MP to standard therapy for remission induction in severe AAV patients may not provide clinical benefit and may be associated with an increased incidence of infections and diabetes.
Fenoglio et al. [11]	The goal should be to develop new approaches that avoid the toxicity associated with currently used agents. These methods should, ideally, be GCs-free.
Neumann et al. [7]	Rituximab is a good substitute for CYC, especially for relapsing and PR3-ANCA positive disease. Reduced GCs dose is possible in ANCA vasculitis attributable to emerging combination induction treatments and alternative medicines.
Floyd et al. [21]	GCs are still the most common supplementary immunosuppression for AAV treatment; however, the narrow therapeutic window necessitates the use of GCs-free therapies.
Monti et al. [12]	The effectiveness and safety of an interleukin-5 inhibitor, mepolizumab, has improved the therapy of refractory or recurrent EGPA.
Samman et al. [9]	If Avacopan was found to be safe and cost-effective, it could be used instead of GCs, leading to a GCs-free AAV regimen. In most patients with severe GPA and MPA, PLEX is no longer regularly recommended; it has not been shown to improve mortality or reduce the risk of ESRD in patients with severe illness. In carefully selected patients, PLEX can still be explored in conjunction with professional recommendations on vasculitis. If rituximab is unavailable or contraindicated, azathioprine and methotrexate should be considered.
Onuora et al. [8]	Patients with AAV could be efficiently treated with the C5a receptor antagonist avacopan as an alternative to GCs, according to the results of the phase III ADVOCATE trial.
Jain et al. [6]	Rituximab has received much interest as a single drug and in combination, and additional trials are underway that could alter the outlook in the next decade.
Anders et al. [22]	The novel study design allowed avacopan to show not only noninferiority to steroids but also superiority to conventional steroid dose, increasing the chances of a successful outcome.

Table 3 below summarizes the characteristics and findings of the observational and experimental studies included in this review [5,10,13-14,21].

**Table 3:** Highlights of the Observational and Experimental Studies HR: Hazard ratio; ANCA: Anti-neutrophil cytoplasmic antibody; MPO-ANCA: Myeloperoxidase- ANCA; PLEX: Plasma exchange; PR3-ANCA: Proteinase 3– ANCA; RPGN: Rapidly progressive glomerulonephritis; ESRD: End-stage renal disease; MP: Methylprednisolone; EUVAS: European vasculitis study group; PLEX: Plasma exchange; IV: Intravenous; CYC: Cyclophosphamide; BVAS: Birmingham Vasculitis Activity Score; CRP: C-reactive protein; RITUXVAS: Rituximab versus cyclophosphamide in ANCA associated vasculitis; GCs: Glucocorticoids; GTI: Glucocorticoid toxicity index

Author	Subjects	Findings
Cortazar et al. [10]	Total: 129	MPO-ANCA was found in 70% of patients, and relapse disease was found in 9%. PLEX was used to treat around 30% of both MPO- ANCA and PR3-ANCA patients. Patients with PR3-ANCA (72%) had considerably higher ear, nose, and throat involvement than those with MPO-ANCA (37%) (p<0.001). RPGN was found in 61% of MPO-ANCA patients and 51% of PR3-ANCA patients, respectively.
McAdoo et al. [13]	Control: 198; Test: 66	By six months, 94% of the patients had achieved disease remission, and at five years, patient and renal survival rates were 84% and 95%, respectively. At two years, 84% were ANCA-negative, and 57% were B cell-depleted, which was associated with a low probability of severe recurrence. During long-term follow-up, the severe infection rate was 1.24 per 10 patient-years. Treatment with the proposed regimen (oral corticosteroids, rituximab, and low-dose pulsed intravenous CYC followed by a maintenance regimen of azathioprine and tapered steroid) was associated with a lower risk of death (HR 0.29; p=0.004), progression to ESRD (HR 0.20; p=0.007), and relapse (HR 0.49; p=0.04)
Pepper et al. [14]	Control: 172; Test: 49	Creatinine, proteinuria, CRP, ANCA level, and BVAS level decreased in the test group patients, indicating remission. Three patients who required dialysis at the time of presentation became dialysis-free. For the treatment of vasculitis, two patients required the additional maintenance of GCs. In reference to the RITUXVAS study, overall results were comparable to matched cohorts from prior EUVAS trials but with lower total exposure to CYC and GCs (p<0.001) and lower rates of severe infections (p=0.02). In the first year, no new cases of diabetes were identified, compared to 8.2% in the EUVAS trials (p=0.04).
Chanouzas et al. [5]	Total: 114; Control: 62; Test: 52	After controlling for confounding factors, MP treatment was related to a higher risk of infection during the first three months (HR 2.7; p=0.004) and a higher incidence of diabetes (HR 6.33; p=0.002).
Floyd et al. [21]	Total: 43; PLEX: 12; IV-MP: 16; CYC: 39; Rituximab: 4	GCs were used in combination with CYC or rituximab in the therapy regimens. The relationship between cumulative GCs dosages and GTI scores was statistically significant (p=0.008). Mood instability and GCs-induced psychosis manifested earlier than adrenal insufficiency, which typically emerged later in the follow-up. The occurrence of infection-related adverse events was consistent throughout the study.

## Review

This section discusses the prevalence and significance of AAV, including disease pathology, traditional treatment modalities/advancements in treatment modalities, especially the combination therapy, while also discussing the limitations of this study.

Prevalence and significance

The diagnosis and treatment of AAV have a brief history in the medical field, where the first association between ANCA and vasculitis was made in 1982, with a report detailing eight patients sharing similar clinical presentations [18]. The emergence of AAV is relatively uncommon, for example, in a general care setting with 8000 patients, one new case emerges every five years [23] with an estimated prevalence of 200-400 cases per million people globally [3]. Recent data estimated 20 cases per million people in North America [1] and approximately 20-25 cases per million in Europe [23]. AAV has been documented to affect men and women equally; however, there are distinctions between younger and older patients, with the former being largely affected by eosinophilic granulomatosis with polyangiitis (EGPA) and the latter by granulomatosis with polyangiitis (GPA) and microscopic polyangiitis (MPA) [3]. Moreover, according to the European League Against Rheumatism (EULAR), the five-year survival rate is estimated between 74% and 97% depending on whether the AAV has connected to GPA, MPA, and EGPA, also known as Churg-Strauss syndrome [2-3]. The prognosis has only recently improved due to advancements in therapeutic approaches [3], eventually leading to favorable prognosis and remission rates.

Disease pathology

Vasculitis can present in various forms [1-2] where medium or large vessels may be impacted depending on the subtype of vasculitis. However, AAV affects the small vessels in response to the immune system attacking neutrophils leading to the production of autoantibodies [1]. Geetha and Jefferson (2020) noted that the condition might present in three distinct forms: MPA, GPA or Wegener, and EGPA or Churg-Strauss [1]. There is limited understanding of the cause and pathology of the condition, except for the toll-like receptor 3 (TLR3) role. This receptor's activation was previously linked to immunological complex glomerular disease and viral/toxin-induced glomerulonephritis. Proteinase 3, myeloperoxidase, elastase, cathepsin G, lactoferrin, and lysozyme are intracellular proteins that ANCAs target in granulocytes. With a high degree of sensitivity and specificity, the P-ANCA and C-ANCA patterns match anti-MPO (97%) and anti-PR3 (80%) [19].

The clinical presentation often impacts the pulmonary-renal system, as seen with pulmonary hemorrhage and hematuria. The constitutional symptoms of AAV include fever, weight loss, myalgia, fatigue, night sweats, polyarthralgia, muscle aches, weakness, and sinus symptoms [23], in addition to the cardiac and gastrointestinal symptoms that may be experienced in some patients [19]. These symptoms ultimately signify chronic inflammation including pulmonary, and renal abnormalities indicative of worsening AAV [23].

Traditional treatment and management modalities

Traditionally, GCs with CYC were considered the first-line treatment for AAV due to their overall effectiveness in inducing remission [4]. Prior to the employment of the combined treatment option, the mortality rate was as high as 80% within a year of diagnosis [9]. High doses of GCs were often provided to induce the remission state initially and were later tapered down to achieve effective maintenance of the condition. However, the use of GCs, particularly in high doses, resulted in undesirable adverse effects, including osteoporosis, diabetes, increased risk of infections, glucose-induced psychosis, and progressive organ damage [7,22] that was measured by the glucocorticoid toxicity index (GTI) [21]. Alternatively, the use of CYC has successfully achieved remission in 75% to 90% of patients [9] when treated with a combined regime with medications such as GCs or rituximab [4,6]. However, CYC is also associated with several adverse effects, including urotoxicity, hematologic toxicity, infertility, cystitis, transitional-cell cancer of the bladder, and an increased risk of infections [6], hence warranting new treatment modalities.

Advancements in treatment and management modalities

In recent decades, new studies, trials, and pharmaceutical developments have created and employed new treatment and management modalities. GCs and CYC are often still employed despite advancements, though they may be combined with new treatment modalities [2,9-15]. Prior to discussing combined techniques, Table 4 will address the rationale of various advanced treatment modalities [2,4-9,15].

**Table 4:** Comparisons Between the Advancements in Treatment and Management Modalities CYC: Cyclophosphamide; GPA: granulomatosis with polyangiitis; MPA: Microscopic polyangiitis; EGPA: Eosinophilic granulomatosis with polyangiitis; AAV: ANCA-associated vasculitis; C4aR: C5a receptor; GCs: Glucocorticoids; ESRD: End-stage renal disease; BAFF: B cell-activating factor; BLyS: B lymphocyte stimulator; IV: Intravenous

Treatment modality	Rationale for the purposed treatment modality
Rituximab [2,6-7,9]	Higher effectiveness than that of CYC. Addresses the issues of relapse commonly associated with CYC. Suggested as the first-line treatment due to its high level of safety and overall success in attaining remission in patients with a level of evidence with GPA and MPA. In patients with EGPA, the level of evidence for rituximab was lower than in GPA/MPA, as evidenced by a retrospective analysis in 41 patients with EGPA.
Avacopan [8]	Superior in the tapering process and in achieving sustained remission after a 52-week follow-up. Demonstrated benefits in shifting the albuminuria and the estimated glomerular filtration rates in patients with AAV. Improvements in kidney function have also been noted. In summary, as a C5aR antagonist, Avacopan offers a unique alternative to GCs.
Plasma Exchange [6,9,15]	A relatively new treatment that remains controversial in contemporary usages. The process involves removing, treating, and returning a "purified" form of blood plasma similar to that of a renal dialysis process. Samman et al. noted that plasma exchange had reduced the need for dialysis at three- and 12-month follow-up. Another study by Jain et al. noted a reduced progression to ESRD because of plasma exchange. This treatment is considered revolutionary for patients with severe acute renal damage where other medications have failed.
Immunological Targeting [4]	In patients with significant granulomatous inflammation immunological targeting has been implemented. Involves the use of Belimumab, tabalumab, or blisibimod, which are designed to target BAFF and/or BLyS. Medications such as abatacept, rituximab, ofatumumab, and ocrelizumab have demonstrated success in targeting CD80/CD8 and CD20 found in T-cells in the body. Though some success has been noted, many of these treatments are still undergoing trials to assess their effectiveness in the immunological targeting process. Their use may be critical in the future.
IV Pulse Methylprednisolone [5]	In severe cases of AAV, IV pulse methylprednisolone is commonly employed. Provides smaller quantities of methylprednisolone to reduce the adverse or toxic side effects. The process often lasts for three days and may be combined with CYC and/or high doses of corticosteroids to achieve remission among severe patients. However, the process lacks sufficient evidence to support its use or demonstrate significant benefits to acute patients. One study recently noted no significant difference in renal recovery, relapse, or survival among severe patients. Instead, the study reported a heightened risk of infection among these patients. Further study is therefore required to assess the effectiveness and safety of this contemporary treatment modality.

Combined treatment modalities

Combined treatment modalities are commonly initiated in the treatment of AAV. These include the use of plasma exchange with GCs, rituximab in combination with GCs, and rituximab in combination with CYC, which is briefly discussed below [9-10,13-15].

Plasma Exchange and GCs

Plasma exchange is not often conducted as a sole treatment option; instead, the employment of plasma exchange is often combined with GCs [9,15]. This is particularly true in cases of severe or acute progression of AAV [15]. One study examined the difference between progression to ESKD and eventual death in patients receiving the combined approach compared with those receiving standard treatment without plasma exchange [9,15]. The results indicated little benefit from the integrated approach, with 100 of 352 patients succumbing to ESKD-related death in the plasma exchange group compared with 109 of 352 in the control group [15]. Further research has been recommended and must be completed to indicate that the combined approach is feasible as an effective treatment and management modality.

Rituximab and GCs

Rituximab and GCs have also been used as a contemporary treatment approach. The goal of the combined approach is to have the ability to decrease the GCs doses, which are accompanied by serious side effects with long-term use. In a randomized controlled trial, one study employed the combined approach, comparing the effectiveness to patients receiving only treatment with high-dose GCs. The patients included were recently diagnosed patients without severe glomerulonephritis or alveolar hemorrhage. The results demonstrated equal levels of success between the treatment modalities, indicating that the combined approach may provide effective remission and treatment without the high-dose GCs [16].

Rituximab and CYC

Another combined treatment option currently employed includes rituximab and CYC [10,13-14]. This approach entirely removes the use of GCs and is presently considered a novel approach to treating AAV [14]. The studies have shown positive results, with benefits such as the reduced risk of death, reduced progression to ESKD, higher rate of achieving remission, and successful rapid tapering from high-dose GCs [10,13-14]. The risk of relapse has also been noted to decrease with the combined approach [13]. The treatment is notably superior among several studies to the standard traditional care provided [10,13-14]. A higher level of attention ought to be provided to future treatment and management efforts.

A note on mepolizumab

The latest evidence of efficacy and safety of an interleukin-5 inhibitor, mepolizumab, has improved the therapy of refractory or recurrent eosinophilic granulomatosis with polyangiitis (EGPA) [12]. A randomized controlled study supports the use of mepolizumab (at a dosage of 300 mg subcutaneously per month) for relapsing or refractory EGPA. The hazard ratio for relapse in favor of mepolizumab was 0.32, with an annualized relapse risk of 50% lower than placebo [20]. Despite the excellent effects of mepolizumab, relapses are still prevalent, and the use of short- and long-term glucocorticoids is common [20]. Further studies are required in order to address these demands and clarify the specific role of mepolizumab.

Limitations

This systematic review's limitation is that it only includes studies from the last five years to focus on the recent therapeutic advancements, and only smaller groups of studies were discussed, excluding any randomized controlled trials in order to avoid contradicting outcomes. The authors believe that more extensive clinical trials in the population of interest could improve remission rates while minimizing adverse effects.

## Conclusions

AAV is a multisystem illness that typically affects the small blood vessels of the renal and respiratory systems, where patients are presently treated with typical immunosuppressive medications that may have a devastating impact on them. Before developing pharmacological treatment and management options, the patient prognosis was often poor, with premature mortality often associated with end-stage renal disease. Given the growing number of targeted therapies accessible, precision medicine in AAV is essential. The traditional use of GCs, usually employed with CYC, has demonstrated success and significantly improved life expectancy. However, high doses of GCs often result in toxicity and a multitude of adverse effects from long-term use. Current treatment and management options seek options free of GCs while retaining the efficacy demonstrated in its ability to provide remission and extend the patient's healthspan.

In conclusion, the most effective treatment modality appears to be the combined approach that includes rituximab and CYC, which offers a safer option than GCs while demonstrating equal, if not better, effectiveness and outcomes. It may also aid in maximizing clinical results while reducing the risk of unwarranted medication toxic effects and expenses. Though several contemporary treatment options are showing promise, including the use of rituximab, additional trials are required to undertake extensive research to find alternative modalities with more favorable outcomes.
